# Cone-Beam Computed Tomography (CBCT)-Guided Non-surgical Management of Type II Dens Invaginatus in Maxillary Lateral Incisors Using Calcium Silicate-Based Materials: A Case Series

**DOI:** 10.7759/cureus.101205

**Published:** 2026-01-09

**Authors:** Prerna Priya

**Affiliations:** 1 Conservative Dentistry and Endodontics, All India Institute of Medical Sciences, Rishikesh, IND; 2 Dentistry, Employees' State Insurance Corporation (ESIC) Medical College and Hospital, Faridabad, IND

**Keywords:** biodentine, calcium silicate–based materials, cone-beam computed tomography, dens invaginatus, periapical lesion

## Abstract

Dens invaginatus (DI) is a developmental anomaly that may create complex internal anatomy, predisposing affected teeth to early pulp necrosis and extensive periapical pathology, particularly in maxillary lateral incisors. Successful management depends on accurate diagnosis, 3D assessment of the invagination, and the use of materials capable of sealing irregular canal spaces and managing open apices or perforations. This report describes the non-surgical endodontic management of two maxillary lateral incisors with Oehlers type II DI associated with necrotic pulp and perforation in the first case and a large periapical lesion in the second case. In both cases, cone-beam computed tomography (CBCT) was used to delineate the extent of the invagination, identify perforations, and evaluate lesion size and proximity to adjacent structures. After careful removal of the invaginated mass and thorough chemomechanical debridement with adjunctive calcium hydroxide medication, treatment strategies were tailored to the root morphology and pathology. In the first case, a coronal perforation was repaired with Biodentine, and the canal was obturated with a calcium silicate-based sealer and gutta-percha. In the second case, an immature tooth with an open apex and extensive periapical lesion was obturated completely with Biodentine. Follow-up periods of 6 and 24 months, respectively, demonstrated satisfactory periapical healing and maintained function, supporting CBCT-guided, bioactive material-based conservative management of complex DI cases.

## Introduction

Dens invaginatus (DI), also known as dens in dente, is a developmental anomaly in which an infolding of the enamel organ into the dental papilla produces a deep enamel-lined invagination within the crown and/or root [1,2]. The resulting distorted internal anatomy predisposes the tooth to early pulpal necrosis and periapical pathology, often in the absence of caries or overt trauma [1,3]. Several proposed etiological factors include trauma, infection, and aberrant growth pressures during odontogenesis; however, none have been definitively established. Reported prevalence ranges from about 0.3% to 10% in different populations, with a marked predilection for maxillary lateral incisors and a frequent association with lingual pits or foramen caecum [1-4].

Oehlers’ classic radiographic classification remains the most widely used, describing three types according to the apico-coronal extent of the invagination and its relationship to the pulp and periodontal ligament (PDL) [5]. In type I, the invagination is confined to the crown; in type II, it extends beyond the cemento-enamel junction as a blind sac; and in type III, it penetrates through the root to communicate with the PDL [5]. More recent authors have refined type III into IIIa (lateral communication with the PDL) and IIIb (apical opening into the periapical tissues), emphasizing the potential for periodontal and periapical disease even in the presence of a vital main canal [6]. Type II and III lesions are particularly challenging because they may present with open apices, perforations, thin radicular walls, and extensive periapical or cyst-like defects [1,6]. Failure to recognize DI on routine radiographs can lead to delayed diagnosis and persistent disease despite previous endodontic treatment [1,2].

Conventional periapical imaging frequently underestimates the true extent and configuration of DI. Cone-beam computed tomography (CBCT) provides 3D visualization of the invagination, the main canal, perforations, and periapical defects. It is now considered invaluable for diagnosis, treatment planning, and follow-up in complex DI cases, with evidence supporting reliable CBCT-based interpretation compared with 2D assessment [6-8]. Recent case reports and series involving Oehlers type IIIa and IIIb DI have shown that CBCT-guided access, combined with the operating microscope, can allow strictly nonsurgical management even when large lesions and aberrant canals are present [9,10].

In parallel, contemporary endodontic literature increasingly supports nonsurgical management of extensive through-and-through periapical lesions [11]. Case series and observational studies report high healing rates for large cyst-like radiolucencies when meticulous chemomechanical debridement is combined with hydraulic calcium-silicate cements or bioceramic sealers, which provide a high pH, sustained calcium release, and bioactivity that favor apatite deposition, cementogenesis, and bone regeneration at the lesion interface [12-16]. Adjunctive use of platelet-rich fibrin (PRF) as an intracanal or intrabony scaffold has been proposed to support healing through sustained growth-factor release and provision of a 3D fibrin matrix [10,17-19]. These data collectively challenge the traditional assumption that large or cortically perforating lesions necessarily require primary surgical intervention, while also underscoring ongoing debate regarding case selection, healing timelines, and indications for surgery [14-16].

Minimally invasive endodontic concepts have evolved alongside these material advances. Ultrasonic tips used under magnification permit conservative refinement of access cavities, selective dentine removal, and targeted debridement of complex anatomy, facilitating irrigant penetration and activation while preserving pericervical dentine [20,21]. When combined with flowable bioceramic sealers and hydraulic cements that can seal irregular spaces and thin-walled roots, such approaches help maintain both the biological seal and the mechanical integrity of endodontically treated teeth [9,10,20,21].

Despite these developments, the evidence base for DI management still consists largely of isolated case reports and small series with heterogeneous protocols and limited long-term follow-up [2,6,11]. Additional well-documented clinical reports using CBCT-guided planning, conservative ultrasonic access, and contemporary bioactive materials are therefore needed to illustrate practical decision-making and medium- to long-term outcomes in anatomically complex type II and III DI affecting maxillary anterior teeth.

## Case presentation

Case 1

**Figure 1:**
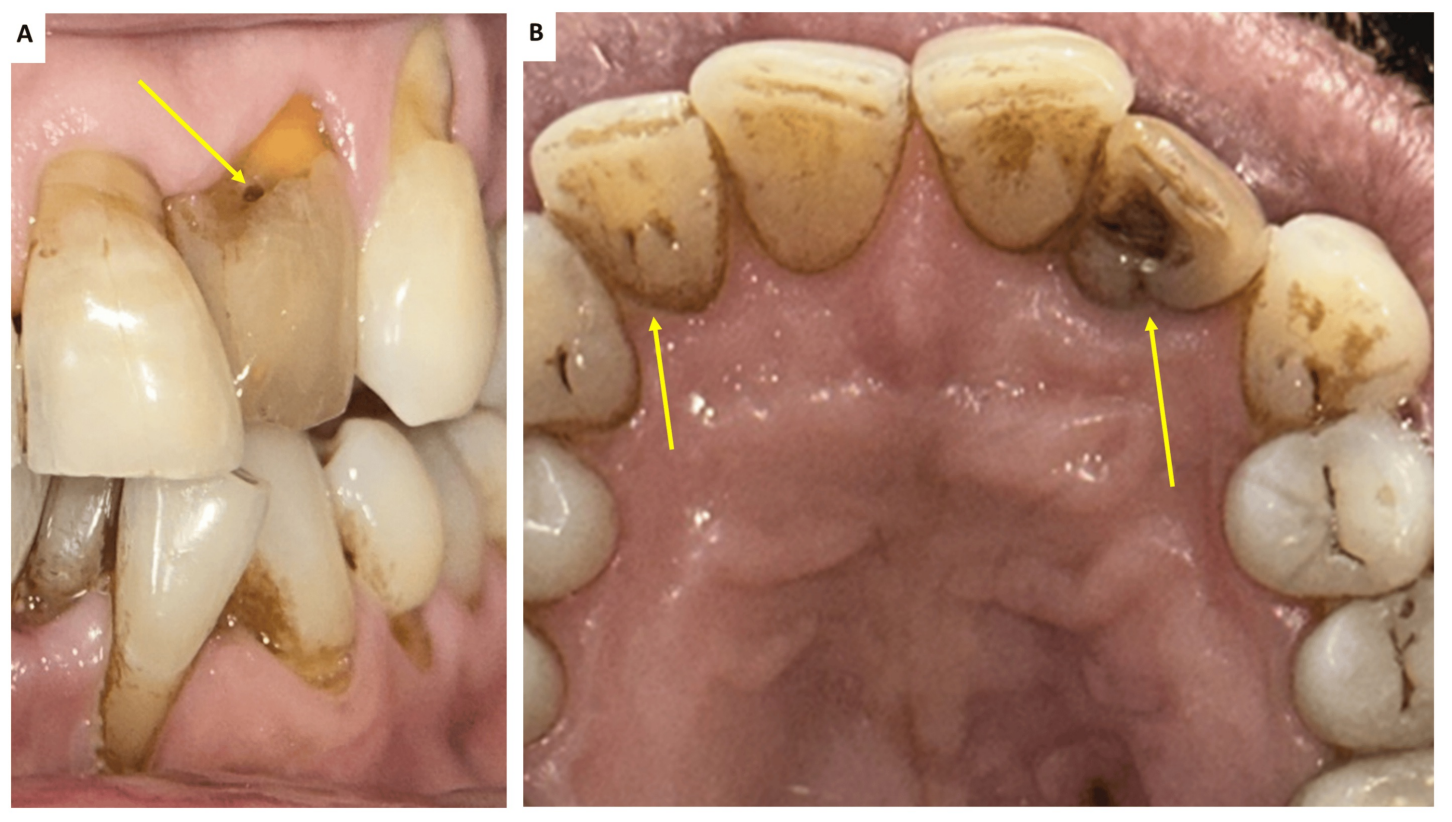
(A) Discolored tooth #22 with labio-incisal perforation in the coronal aspect (indicated by arrow) and (B) clinical photograph of the maxillary anterior teeth with arrows showing palatogingival groove in tooth #12 and #22

A 59-year-old male patient reported to the Department of Dentistry with a chief complaint of swelling in the upper left incisor region. He was concurrently undergoing periodontal treatment for root coverage in the Department of Periodontology. Clinical examination of the maxillary left lateral incisor (#22) revealed grayish discoloration, insensitivity to percussion, and absence of mobility. The palato-gingival groove was examined, and the periodontal probing depths were within normal limits. A previously prepared coronal access cavity into the pulp chamber had been prepared, and the temporary restorative material had been dislodged. The patient had no history of trauma, and his medical history was noncontributory. Clinically, tooth #22 presented a pit on the lingual aspect and was caries-free. Both maxillary lateral incisors had lingual pits, but the right lateral incisor showed no anatomic variations (Figure 1). Based on clinical findings and the presence of a sinus tract, tooth #22 was diagnosed with a chronic apical abscess.

**Figure 2:**
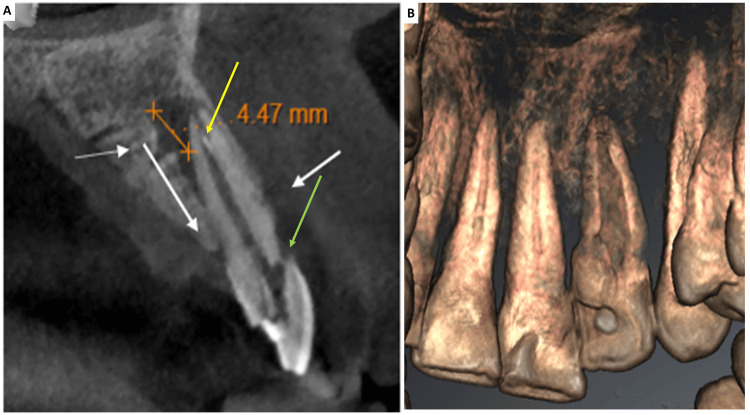
(A) CBCT revealed residual gutta percha fragment (yellow arrow) and a labio-incisal perforation (green arrow) in the coronal aspect and confirmed by (B) 3D reconstruction CBCT: cone-beam computed tomography, 3D: three-dimensional

Periapical radiographic evaluation demonstrated a radiolucent pocket with a radiopaque border extending into the root as a blind sac below the cementoenamel junction, consistent with Oehlers type II DI. Tooth #22, which had been previously treated endodontically, showed remnant gutta-percha material in the canal. Given the complex root canal anatomy observed on the intraoral periapical radiograph, a CBCT scan (Sirona Orthophos S Scanner) was obtained for detailed assessment. CBCT revealed a mixed-density mass within the root canal space and a labio-incisal perforation in the coronal aspect (Figure 2).

**Figure 3:**
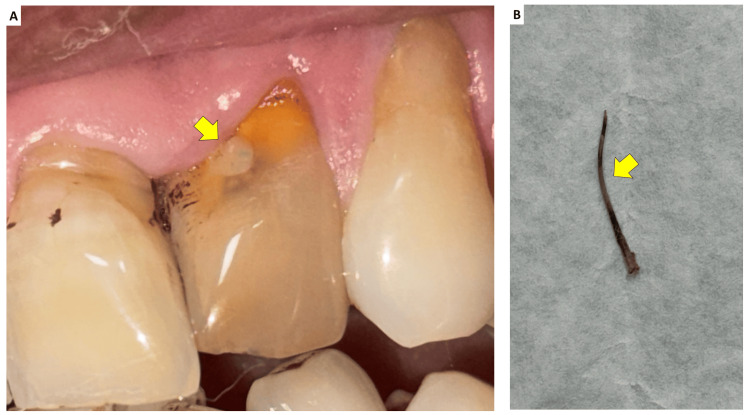
(A) The labio-incisal perforation was repaired using a calcium silicate-based material (Biodentine, Septodont, Saint-Maur-des-Fossés, France) over which a layer of glass ionomer cement was placed and (B) residual gutta-percha retrieved with files

After explaining the treatment options, written informed consent was obtained from the patient. Local anesthesia was achieved with 1.8 mL of 2% lidocaine containing 1:80,000 epinephrine, and rubber dam isolation was performed. Access to the DI was gained through the palatal surface using a round diamond bur (BR-41, DIA Burs, Mani Inc., Japan). After refining the coronal access, a DG-16 endodontic explorer (Hu-Friedy Mfg. Co., Chicago, IL, USA) was used to locate the canal orifices. The labio-incisal perforation was repaired with a calcium silicate-based material (Biodentine, Septodont, Saint-Maur-des-Fossés, France), followed by a layer of glass ionomer cement (Ketac Molar, 3M) (Figure 3).

The intracanal invaginated mass was carefully removed with ultrasonic tips (Woodpecker ED4D Endodontics; Guilin Woodpecker Medical Instrument Co., Ltd, China), and the residual gutta-percha fragment was retrieved with files. Working length was established with a size 15 K-file (Mani, Tochigi, Japan). The internal canal walls were instrumented manually up to size 40 K-file. Irrigation was performed using 5.25% sodium hypochlorite (20 mL; Prime Dental Products Pvt. Ltd., India) delivered with a 27-gauge side-vented needle, alternating with saline. Irrigant activation was carried out using the EndoActivator system (Dentsply Sirona, New York, USA). The smear layer was removed with 2 mL of 17% EDTA for three minutes, followed by a final flush with 3 mL of saline to remove residual chelator.

The canal was dried with paper points (DiaDent, Dia-Pro T Plus, Canada), and a calcium hydroxide paste (Avuecal, Dental Avenue, India) was placed as an intracanal medicament. The access cavity was sealed with a 4-mm-thick layer of Cavit G (3M ESPE), and the patient was scheduled for a two-week recall.

**Figure 4:**
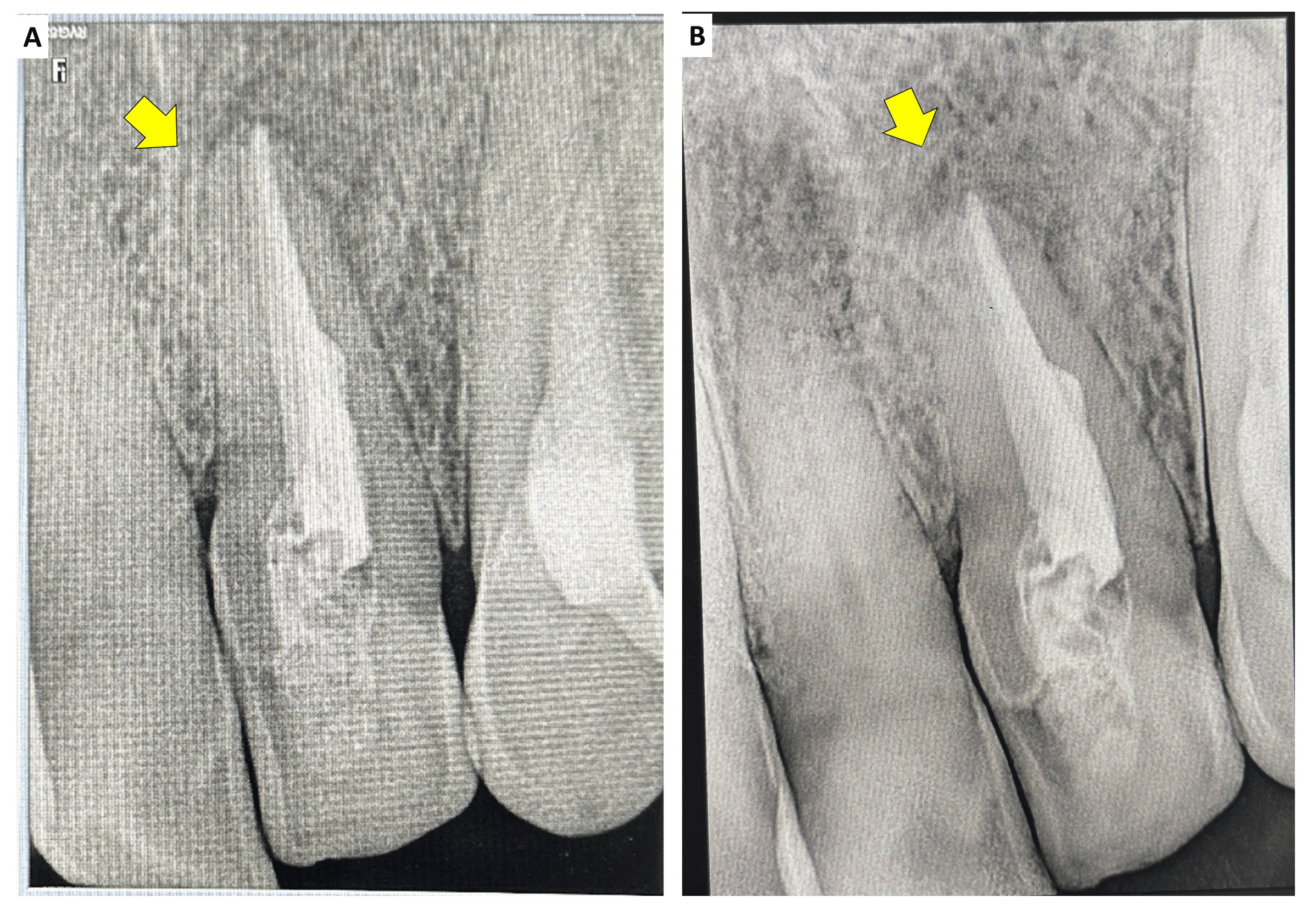
(A) Obturation was performed using a calcium silicate-based sealer and gutta-percha and (B) six months follow-up

At the 14-day follow-up, the patient was asymptomatic. Under rubber dam isolation, the temporary restoration and calcium hydroxide dressing were removed using saline irrigation and a size 40 K-file. The canal was irrigated with 3.0% sodium hypochlorite using a 27-gauge side-vented needle and activated with the EndoActivator. After final drying with paper points, obturation (Figure 4) was performed using a calcium silicate-based sealer (Bio-C Sealer, Angelus, Londrina, PR, Brazil) and gutta-percha cones (Dentsply). A glass ionomer cement barrier was placed in the coronal third of the canal, and the access cavity was permanently restored with composite resin (Tetric N-Ceram, Ivoclar Vivadent, Liechtenstein). At the six-month follow-up, the patient was asymptomatic, with no tenderness to percussion or palpation. Periapical radiographic examination demonstrated satisfactory periapical healing and reduction in radiolucency (Figure 4). The restoration remained intact, and no clinical signs or symptoms of treatment failure were observed. The patient did not experience any problems in masticatory functioning or recurrence of the swelling.

Case 2

**Figure 5:**
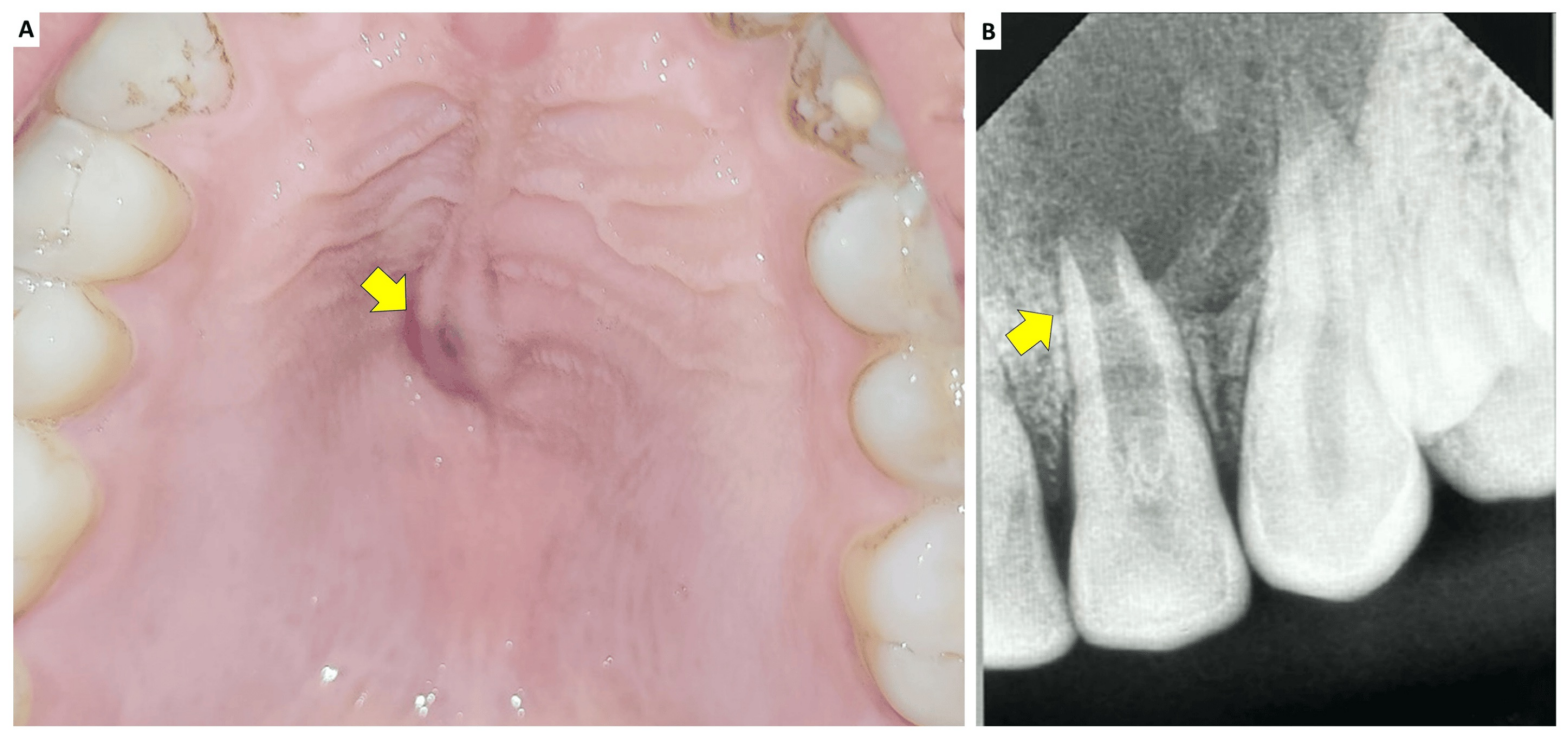
(A) Preoperative images of #22 with palatal non-draining sinus and (B) periapical radiography of tooth #22 showed a radiolucent pocket with a radiopaque border extending into the root as a blind sac up to the cementoenamel junction. A blunderbuss canal #22 and a well-defined periapical radiolucency involving the apices of teeth #21, #22, and #23 were evident

A 22-year-old male patient was referred to the Department of Dentistry with a chief complaint of pus discharge in the upper left anterior region. Clinical examination revealed a palatal non-draining sinus tract in relation to the maxillary anterior teeth. There was no history of trauma, and the medical history was noncontributory. Teeth #21, #22, and #23 demonstrated tenderness to percussion and palpation. Pulp sensibility testing with cold (Endo Ice, Hygenic, Akron, OH, USA) and an electronic pulp tester yielded no response in these teeth. Periapical radiography of tooth #22 showed a radiolucent pocket with a radiopaque border extending into the root as a blind sac up to the cementoenamel junction, consistent with Oehlers type II DI (Figure 5). A blunderbuss canal with tooth #22 and a well-defined periapical radiolucency involving the apices of teeth #21, #22, and #23 were evident.

**Figure 6:**
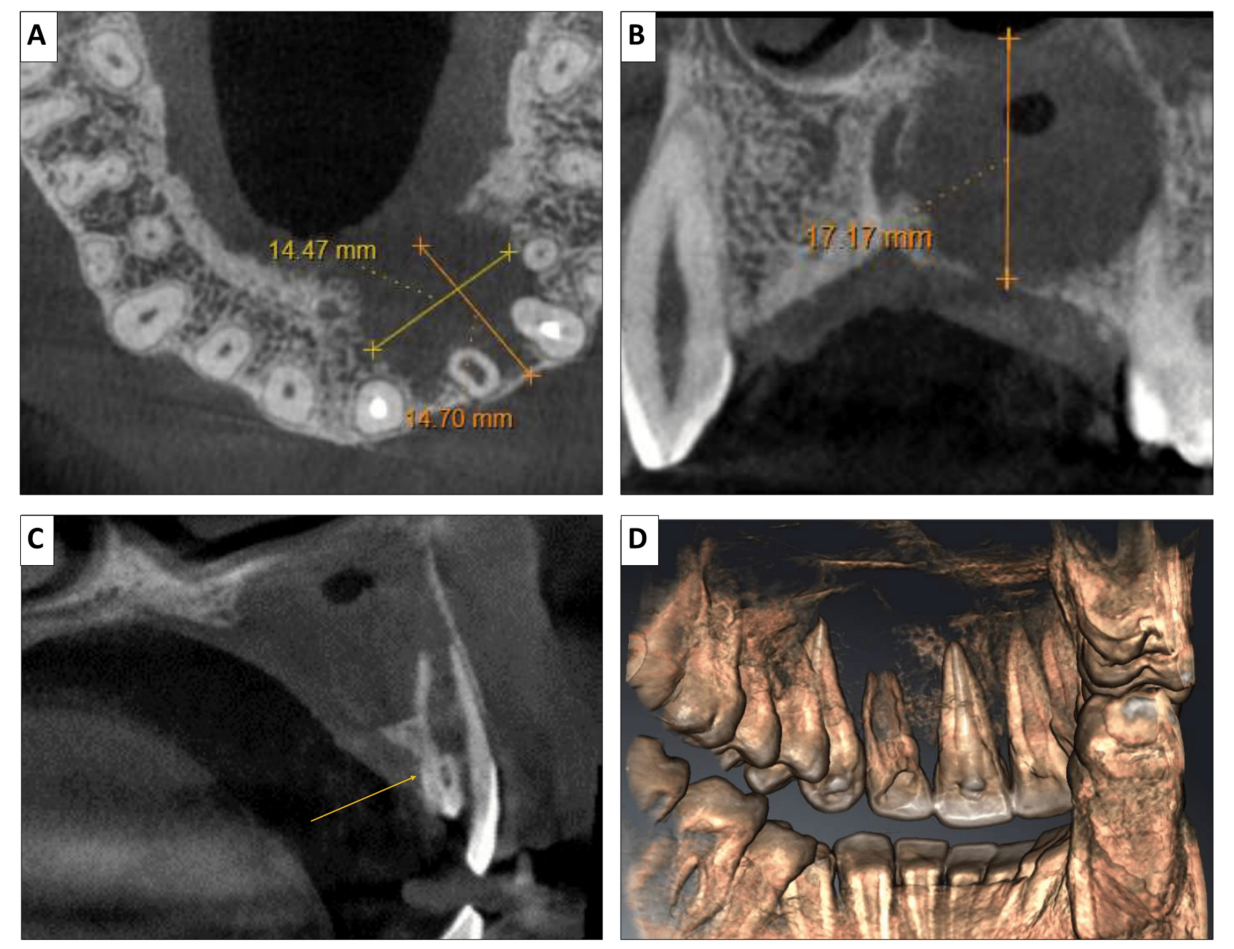
CBCT scans showing well-defined radiolucent lesion with cortication involving the maxillary left central incisor to maxillary first premolar region in (A) axial view, (B) coronal view, (C) sagital view, and (D) 3D reconstruction CBCT: cone-beam computed tomography

Given the complexity of the anatomy, a CBCT scan of the anterior maxilla was obtained. CBCT evaluation revealed a well-defined unilocular radiolucent lesion measuring approximately 23.7 × 19.5 mm in the periapical region of teeth #11, #21, #22, and #23, with its epicenter at the periapical area of #22 (Figure 6). The lesion extended superoinferiorly to approximately 20.5 mm, caused resorption of the nasal floor and medial wall of the maxillary sinus, and showed palatal cortical plate perforation and bone resorption crossing the midline. An open apex was observed in relation to #22, with internal mixed-density areas suggestive of dystrophic calcifications within the lesion.

Based on the history, clinical findings, and imaging, a diagnosis of acute symptomatic apical periodontitis associated with DI in tooth #22 and a large periapical lesion involving teeth #21-23 was established. He had undergone root canal treatment for teeth #21 and #23 at a private clinic during the interim period, and these teeth were no longer tender to percussion. Various treatment options, including surgical and non-surgical approaches, were discussed with the patient, who opted for non-surgical endodontic management. Written informed consent was obtained.

**Figure 7:**
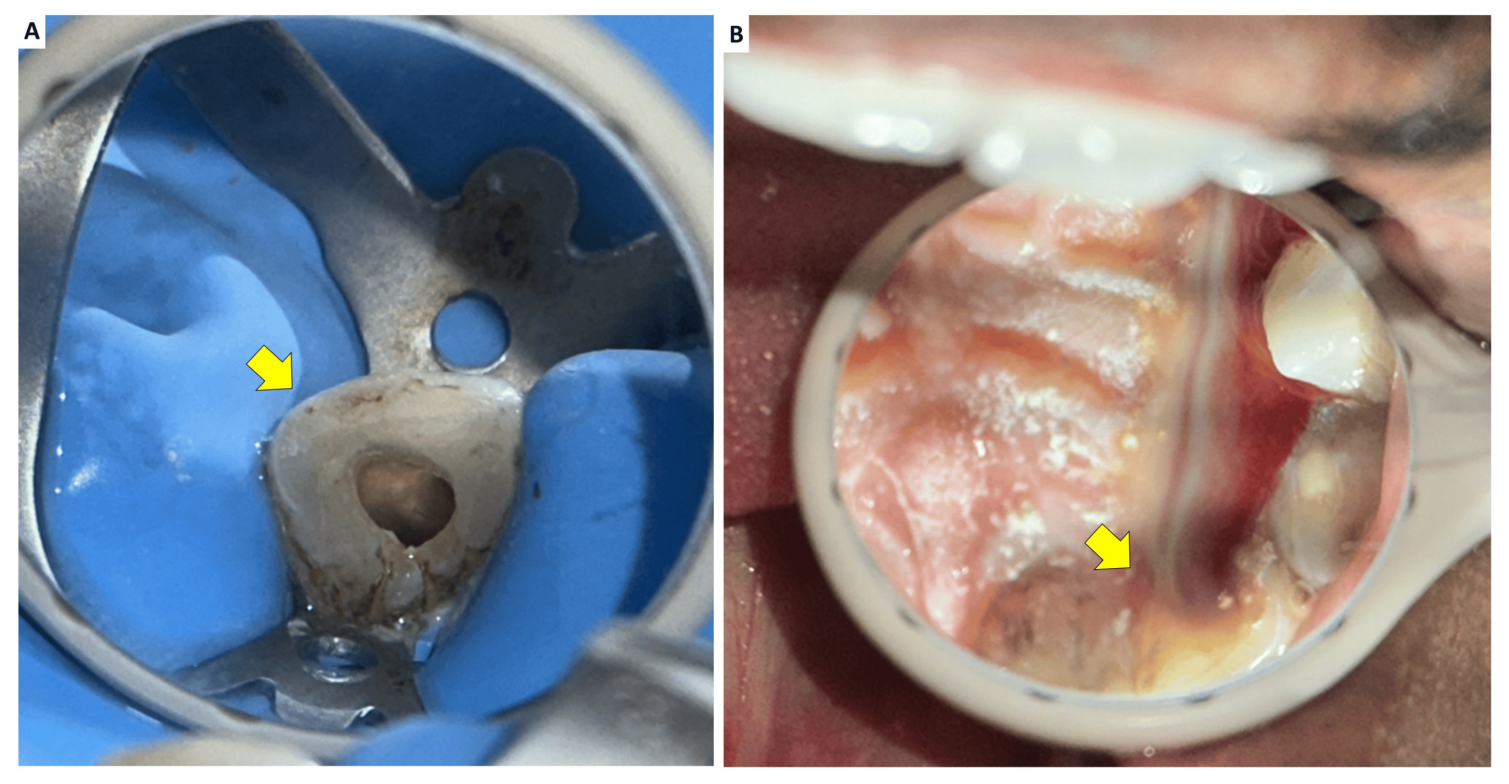
(A) Access to the invaginated canal in tooth #22 was gained from the palatal surface using a round diamond bur and (B) Removal of the temporary restoration resulted in purulent discharge from the canal

At the first visit, local anesthesia was administered using 1.8 mL of 2% lidocaine with 1:80,000 epinephrine, and rubber dam isolation was performed (Figure 7). Access to the invaginated canal in tooth #22 was gained from the palatal surface using a round diamond bur (BR-41, DIA Burs, Mani Inc., Japan). After refining access, a DG-16 endodontic explorer was used to locate the canal orifices. The invaginated mass was carefully removed using ultrasonic tips (Woodpecker Scaler Tip ED4D for DTE and Satelec scalers). Working length was determined with a size 25 K-file (Mani, Tochigi, Japan). Minimal mechanical instrumentation of the canal walls was performed using hand K-files to preserve radicular dentin, particularly given the open apex.

Irrigation was performed using 20 mL of 5.25% sodium hypochlorite (Prime Dental Products Pvt. Ltd., India) and saline delivered via a 27-gauge side-vented needle. Sonic activation of the irrigant was performed with the EndoActivator system. Given the blunderbuss canal and open apex, irrigant delivery and sonic activation were performed using an anti-extrusion protocol. A 27-gauge side-vented needle was used without binding, kept short of the working length, and irrigant was expressed slowly to allow continuous coronal backflow. Sonic activation was performed passively, with the activator tip maintained short of the apical terminus and not engaging the canal walls, using brief activation cycles with replenishment and aspiration between cycles. No apical patency filing or intentional apical enlargement was performed to avoid increasing the risk of irrigant extrusion. The smear layer was removed with 2 mL of 17% EDTA for three minutes, followed by a final saline rinse. The canal was dried with paper points, and calcium hydroxide paste (Avuecal, Dental Avenue, India) was placed as an intracanal medicament. The access cavity was sealed with a 4-mm layer of Cavit G, and the patient was recalled two weeks later.

At the two-week recall, the patient reported severe pain. On examination of tooth #22, removal of the temporary restoration resulted in purulent discharge from the canal. Under rubber dam isolation, the calcium hydroxide dressing was removed using saline irrigation and a size 50 K-file. The canal was reinstrumented minimally and again irrigated with 20 mL of 3.0% sodium hypochlorite using a 27-gauge side-vented needle, and activated with a #15 U-file (Mani, Tochigi, Japan). The canal was dried, and a fresh dressing of calcium hydroxide paste was placed. Temporization was done with Cavit G.

This protocol was repeated every two weeks for approximately three months. Over successive visits, the amount of exudate gradually diminished, and eventually the canal became completely dry and the patient asymptomatic. After confirmation of the absence of clinical signs and symptoms and a dry canal, a decision was made to obturate the canal with a bioactive material suitable for apexification.

Biodentine (Septodont, Saint-Maur-des-Fossés, France) was mixed according to the manufacturer’s instructions and placed incrementally into the canal using a Machtou plugger size #4, keeping the plugger 2 mm short of the radiographic apex to avoid overextension. The entire canal length up to the cementoenamel junction was filled with Biodentine, creating a monoblock obturation and apical barrier. The access was initially sealed with glass ionomer cement, which was subsequently reduced to a 2-mm intra-orifice barrier. A definitive composite restoration (Tetric N-Ceram, Ivoclar Vivadent, Liechtenstein) was then placed.

**Figure 8:**
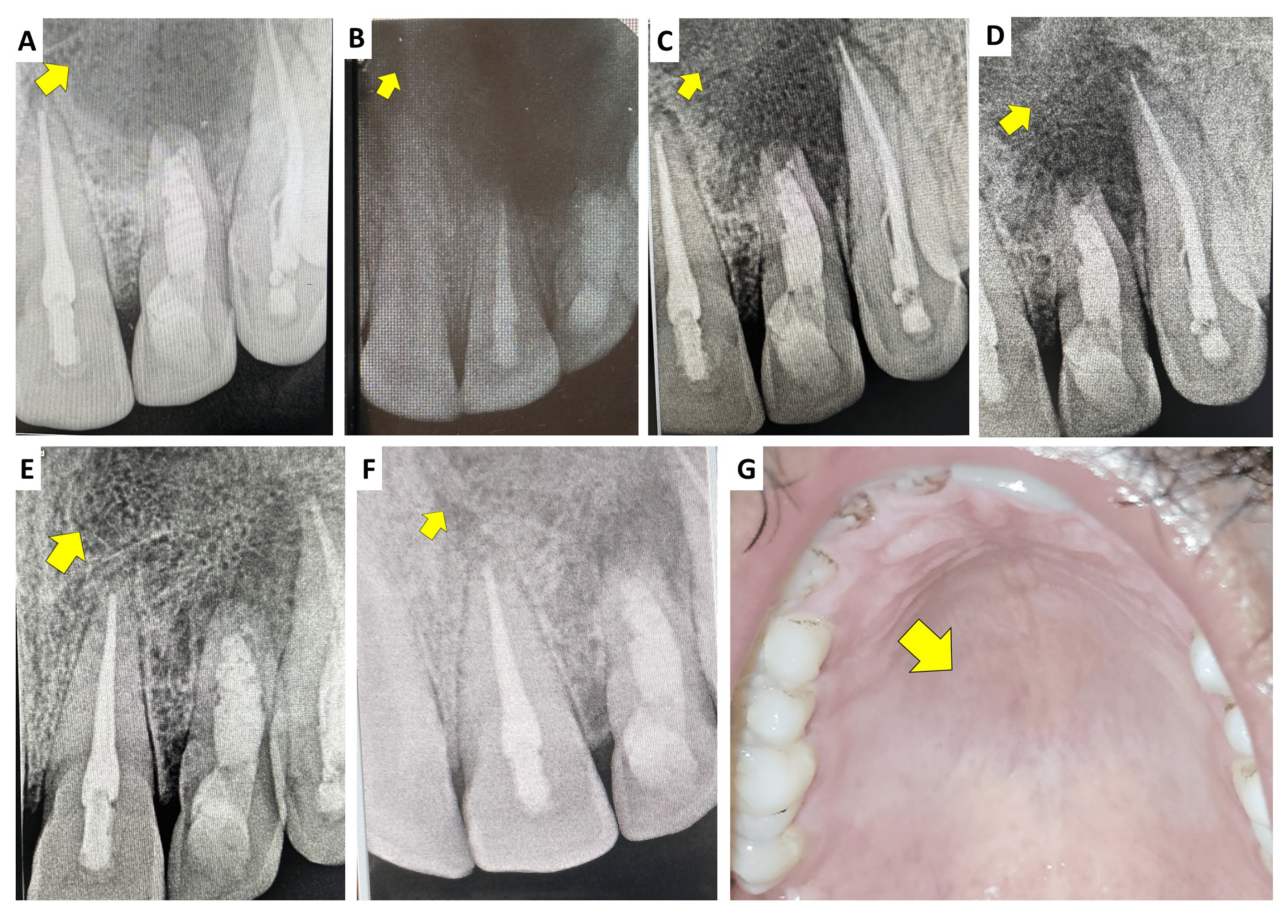
(A) Immediate postoperative radiograph after obturation with Biodentine and follow-up radiographs after (B) three months, (C) six months, (D) 12 months, (E) 18 months, and (F) 24 months and (G) clinically healed lesion at 24 months

At the one-month post-obturation follow-up, the patient was asymptomatic, and the palatal sinus tract had resolved. Subsequent recalls at 3, 6, 12, 18, and 24 months showed progressive radiographic evidence of periapical healing (Figure 8), with marked reduction in lesion size, reformation of lamina dura, and evidence of osteoblastic activity around the periapical region. The restorations remained intact, and teeth #21-23 were asymptomatic, with no tenderness to percussion or palpation. At the 24-month follow-up, the lesion demonstrated advanced radiographic healing, and there were no clinical signs or symptoms suggestive of treatment failure. Functionally, the patient reported comfortable incising/chewing with the anterior segment and satisfaction with the clinical outcome; no swelling, tenderness, or recurrence of sinus tract was reported during follow-up.

An overall summary of both cases to facilitate cross-case comparison is provided in Table 1.

**Table 1:** Summary of clinical presentation, CBCT findings, treatment protocol, and outcomes CBCT: cone-beam computed tomography, DI: dens invaginatus, NaOCl: sodium hypochlorite, EDTA: ethylenediaminetetraacetic acid, GIC: glass ionomer cement, CEJ: cemento-enamel junction

Case	Age/sex	Tooth	Diagnosis	Key CBCT findings	Irrigation/activation (concentration, volume, activation method)	Medicament	Obturation material	Follow-up	Outcome
1	59/M	Maxillary left lateral incisor (#22)	Chronic apical abscess associated with Oehlers type II DI; previously treated tooth with persistent infection	Type II DI with mixed-density mass within canal space; residual gutta-percha fragment; labio-incisal coronal perforation confirmed on CBCT and 3D reconstruction	5.25% NaOCl (20 mL) with 27G side-vented needle + saline (alternating); EndoActivator sonic activation; smear layer removal with 17% EDTA (2 mL for 3 min) followed by final saline flush	Calcium hydroxide paste (Avuecal) for 2 weeks	Coronal perforation repair with Biodentine; obturation with Bio-C Sealer + gutta-percha; GIC intraorifice barrier; composite (Tetric N-Ceram)	6 months	Asymptomatic; radiographic reduction in periapical radiolucency with satisfactory healing; restoration intact; no clinical signs of failure
2	22/M	Maxillary left lateral incisor (#22)	Acute symptomatic apical periodontitis associated with Oehlers type II DI and immature open apex; large periapical lesion involving adjacent teeth (#21–#23)	Well-defined unilocular periapical radiolucency (approx. 23.7 × 19.5 mm; superoinferior extent ~20.5 mm) with epicenter at #22; palatal cortical plate perforation and bone resorption crossing midline; resorption of nasal floor and medial wall of maxillary sinus; open apex; internal mixed-density areas suggestive of dystrophic calcifications	5.25% NaOCl (20 mL) + saline with 27G side-vented needle; EndoActivator sonic activation; smear layer removal with 17% EDTA (2 mL for 3 min) and final saline rinse; during recall visits, canal was irrigated with 3.0% NaOCl and activated using a #15 U-file (Mani) (repeated at ~2-week intervals until canal became dry)	Calcium hydroxide paste (Avuecal), renewed every 2 weeks for ~3 months until the exudate resolves	Full-length obturation with Biodentine (monoblock fill to CEJ) placed incrementally; GIC intraorifice barrier; composite (Tetric N-Ceram)	1, 3, 6, 12, 18, and 24 months	Sinus tract resolved by 1 month; progressive radiographic healing with marked lesion reduction, reformation of lamina dura and osteoblastic activity; asymptomatic at 24 months; restorations intact; no signs of failure

## Discussion

DI is recognized as a developmental anomaly with highly variable internal morphology, and its clinical behavior is strongly influenced by the type and extent of the invagination [1,2,11]. Maxillary lateral incisors are the most frequently affected teeth, and type II and III (including IIIa and IIIb) variants show the highest risk of pulpal necrosis and periapical pathology even in clinically intact or caries-free crowns [1-4,11-13]. Both teeth in the present report were maxillary lateral incisors with Oehlers type II DI and necrotic pulps, fitting well within this established clinicoradiographic profile [1-4,11-13].

A central challenge in DI management is accurately delineating the invaginated complex canal system. Conventional periapical radiographs provide only a 2D projection and may fail to reveal the course of the invagination, the presence of perforations, or the full extent of associated lesions [1,2,6]. In both cases, CBCT was crucial for understanding the 3D relationship between the invagination and the main canal, confirming that the invagination was not fused with the radicular dentine, and identifying a labio-incisal perforation in the first case and characterizing the large lesion with a cortical perforation and sinus involvement in the second. This experience corroborates earlier work emphasizing CBCT as an indispensable adjunct in DI, enabling conservative yet strategically directed access, targeted debridement, and more accurate risk assessment in type II and III cases [6-8,12,13].

Treatment options for DI range from preventive sealing of deep pits in vital teeth to complex non-surgical endodontic procedures, surgical endodontics, intentional replantation, or extraction in advanced cases [1,2,6,11]. Current reviews advocate a tooth-preserving, primarily non-surgical strategy whenever adequate debridement and 3D sealing can be achieved, particularly when guided by CBCT and supported by modern bioceramic materials [6,11-13]. In the present report, CBCT enabled careful removal of the DI using diamond-coated ultrasonic tips under magnification, thereby allowing thorough debridement of the main canal while preserving as much radicular dentine as possible. Retaining the invagination might theoretically contribute to root strength, but treating the invagination and the primary canal independently is complex and may adversely affect the prognosis [2,6,11-13]. A recent clinical trial reported that the efficiency of root canal cleaning increases with a higher NaOCl concentration (5.25%) and is further enhanced by NaOCl activation [14]. The selective ultrasonic approach used here aligns with minimally invasive endodontic concepts that aim to preserve pericervical dentine, maintain mechanical integrity, and still allow effective irrigation and obturation [20,21].

The first case illustrates persistent pathology after previous root canal treatment in which the underlying DI and coronal perforation had likely gone unrecognized. Once the invaginated mass and residual gutta-percha were removed, perforation repair with Biodentine, followed by obturation with a calcium silicate-based sealer and placement of a glass ionomer intraorifice barrier, was chosen to maximize sealing of all possible communication pathways. Biodentine, a hydraulic calcium silicate cement, exhibits favorable sealing ability, high biocompatibility, alkaline pH, and bioactivity, promoting hard-tissue deposition and cementum-like repair in perforation and apexification procedures [9,10]. In both cases, the uneventful clinical course and radiographic healing at six-month follow-up in these structurally compromised teeth support its use in DI cases with thin radicular walls and perforations [9,10].

The second case posed additional difficulties due to an immature root with an open apex and a large lesion extending to adjacent teeth. According to a systematic review and meta-analysis, sonic and ultrasonic activation cause less extrusion [22]. Preventing irrigant leakage/extrusion during sonic activation in open-apex teeth requires a multifaceted approach. These include limiting activation depth, selecting appropriate activation systems with lower extrusion risks, and ensuring controlled irrigant delivery and working length management [23]. Following this protocol adequately in the present case minimized the risk of apical extrusion of the irrigant and consequent sodium hypochlorite accident.

Traditionally, long-term calcium hydroxide apexification was the treatment of choice, but this approach is associated with prolonged therapy, multiple visits, and a potential weakening of root dentine [1,6]. A staged protocol was therefore adopted: calcium hydroxide dressings were used for several weeks to reduce microbial load and control exudation, followed by complete obturation with Biodentine once the canal was dry and asymptomatic. Calcium silicate-based materials have been successfully used as apical barriers or bulk fillings in immature teeth, providing predictable periapical healing and root-end closure in shorter timeframes than conventional apexification, while also potentially reinforcing the remaining radicular dentine [9,10]. The 24-month follow-up in this case, showing advanced radiographic repair and complete sinus resolution, is consistent with these reports and underscores the potential of Biodentine as a monoblock obturation material in carefully selected DI cases with open apices [9,10].

Recent literature further supports non-surgical management as a viable first-line approach for extensive, even through-and-through, periapical lesions. Case reports and retrospective studies have demonstrated high healing rates for large cyst-like radiolucencies managed exclusively with chemomechanical debridement, intracanal medication, and obturation using bioactive hydraulic cements or bioceramic sealers, with CBCT documenting progressive bone fill and cortical regeneration [15-17]. Although surgical enucleation traditionally has been recommended for very large lesions or those with persistent symptoms, these data suggest that, in many cases, non-surgical root canal treatment alone can achieve comparable radiographic and clinical outcomes with lower morbidity [15-17]. The proposed mechanism of non-surgical healing involves the elimination of intracanal irritants, followed by the bioactive material providing a stable, alkaline, calcium-rich interface that favors apatite precipitation, cementogenesis, and osteogenesis along the root surface [9,10,15-17]. Where used, adjuncts such as platelet-rich fibrin can further enhance repair by delivering a fibrin scaffold and growth factors that promote angiogenesis and bone formation, particularly in immature teeth and extensive defects [10,18-20].

In both cases, minimally invasive access strategies were combined with bioactive calcium silicate-based materials and a direct composite coronal restoration to preserve pericervical dentin. The use of diamond-coated ultrasonic tips allowed precise removal of the invagination and refinement of access without unnecessary sacrifice of pericervical dentine, in line with contemporary recommendations for minimally invasive access cavities [21,24]. Direct composite restoration was preferred over indirect full-coverage options because the residual dentine thickness, particularly in the cervical region, was limited, and further tooth reduction would have compromised structural integrity.

In the first case, although non-vital bleaching was planned to address the greyish discoloration, it was deferred because the patient had to travel and preferred to postpone any additional elective procedures. At follow-up, both teeth remained functional, with intact restorations and radiographic evidence of sustained healing.

Taken together, these cases reinforce three key messages for clinical practice: clinicians should maintain a high index of suspicion for DI in maxillary lateral incisors with unexplained or recurrent periapical disease; CBCT should be strongly considered when complex anatomy or extensive lesions are suspected; and contemporary calcium silicate-based materials, used within a systematic disinfection protocol, can enable reliable healing outcomes in challenging DI presentations historically considered candidates for surgical intervention or extraction.

## Conclusions

The present case series demonstrates that a conservative, CBCT-guided assessment combined with bioceramic materials can successfully manage anatomically challenging dens in dente without the need for surgical intervention. Despite a coronal perforation in one case and an open apex with an extensive through-and-through periapical lesion in the other, both teeth remained asymptomatic, with radiographic evidence of progressive bone healing and functional stability during follow-up. Additionally, the use of instruments such as ultrasonics in conjunction with bioceramic materials helps minimize tooth structure loss and maintain the biological and mechanical integrity of the endodontically treated tooth. And hence, such complex treatment could be feasible nonsurgically with a favorable outcome. These findings underscore the importance of accurate 3D diagnosis, meticulous disinfection, and the biological advantages of modern bioactive materials in achieving long-term success. Further well-controlled clinical studies with larger sample sizes and longer follow-up are necessary to optimize treatment protocols and validate outcomes in broader clinical scenarios.
